# Assessment of the regional distribution of normalized circumferential strain in the thoracic and abdominal aorta using DENSE cardiovascular magnetic resonance

**DOI:** 10.1186/s12968-019-0565-0

**Published:** 2019-09-16

**Authors:** John S. Wilson, W. Robert Taylor, John Oshinski

**Affiliations:** 1 Department of Biomedical Engineering and Pauley Heart Center, Virginia Commonwealth University, P.O. Box 980335, Richmond, VA USA; 2 Department of Radiology and Imaging Sciences, Emory University School of Medicine, Atlanta, GA USA; 3 Department of Biomedical Engineering, Emory University and Georgia Institute of Technology, Atlanta, GA USA; 4 Division of Cardiology, Department of Medicine, Emory University School of Medicine, Atlanta, GA USA; 5 Division of Cardiology, Department of Medicine, Atlanta VA Medical Center, Decatur, GA USA

**Keywords:** Aortic mechanics, Strain distribution, Cardiovascular magnetic resonance imaging, DENSE MRI

## Abstract

**Background:**

Displacement Encoding with Stimulated Echoes (DENSE) cardiovascular magnetic resonance (CMR) of the aortic wall offers the potential to improve patient-specific diagnostics and prognostics of diverse aortopathies by quantifying regionally heterogeneous aortic wall strain in vivo. However, before regional mapping of strain can be used to clinically assess aortic pathology, an evaluation of the natural variation of normal regional aortic kinematics is required.

**Method:**

Aortic spiral cine DENSE CMR was performed at 3 T in 30 healthy adult subjects (range 18 to 65 years) at one or more axial locations that are at high risk for aortic aneurysm or dissection: the infrarenal abdominal aorta (IAA, *n* = 11), mid-descending thoracic aorta (DTA, *n* = 17), and/or distal aortic arch (DAA, *n* = 11). After implementing custom noise-reduction techniques, regional circumferential Green strain of the aortic wall was calculated across 16 sectors around the aortic circumference at each location and normalized by the mean circumferential strain for comparison between individuals.

**Results:**

The distribution of normalized circumferential strain (NCS) was heterogeneous for all locations evaluated. Despite large differences in mean strain between subjects, comparisons of NCS revealed consistent patterns of strain distribution for similar groupings of patients by axial location, age, and/or mean displacement angle. NCS at local systole was greatest in the lateral/posterolateral walls in the IAAs (1.47 ± 0.27), medial wall in anteriorly displacing DTAs (1.28 ± 0.20), lateral wall in posteriorly displacing DTAs (1.29 ± 0.29), superior curvature in DAAs < 50 years-old (1.93 ± 0.22), and medial wall in DAAs > 50 years (2.29 ± 0.58). The distribution of strain was strongly influenced by the location of the vertebra and other surrounding structures unique to each location.

**Conclusions:**

Regional in vivo circumferential strain in the adult aorta is unique to each axial location and heterogeneous around its circumference, but can be grouped into consistent patterns defined by basic patient-specific metrics following normalization. The heterogeneous strain distributions unique to each group may be due to local peri-aortic constraints (particularly at the aorto-vertebral interface), heterogeneous material properties, and/or heterogeneous flow patterns. These results must be carefully considered in future studies seeking to clinically interpret or computationally model patient-specific aortic kinematics.

## Background

The fundamental function of the aorta is straightforward: to deliver oxygenated blood from the heart to the body. Nevertheless, the aorta is far from a simple homogeneous tube; it is a continuously remodeling living structure with regionally heterogeneous embryonic origins, geometry, and mechanobiological properties that must adapt to both short-term changes in cardiac output and long-term alterations due to aging and pathology. Compromise of efficient aortic function (e.g., due to aneurysms, dissections, hypertension, or stenoses) can cause potentially fatal primary aortic complications, such as aortic rupture, as well as contribute to serious secondary complications, such as heart failure and malperfusion of end-organs. The unique regional and patient-specific properties of each aorta reflect its proper (or pathologic) function as a mechanical conduit. Thus, development of a reliable, non-invasive method to quantify regional aortic mechanics has great potential for improving patient-specific diagnostics, risk-assessment, treatment planning, and the monitoring of therapeutic efficacy.

The majority of prior in vivo assessments of aortic mechanical properties has focused on metrics that provide only a single value for the entire aorta (e.g., classic pulse wave velocity (PWV) or cardio-ankle vascular index (CAVI) [1]) or homogeneous values at different axial locations along the aorta (e.g., local PWV by 4D flow cardiovascular magnetic resonance (CMR) [2] or circumferential distensibility calculated by cine ultrasound, computed tomography (CT), or CMR [3]). These methods provide a measure that correlates to aortic wall stiffness; however, single value metrics like PWV cannot distinguish heterogeneities in aortic properties along the length of the aorta, and cross-sectional homogenized values cannot identify circumferential heterogeneities around the aorta at a given axial location. Notably, many critical aortic pathologies, such as aneurysms and dissections, are asymmetric in nature both circumferentially and longitudinally, and aortic rupture is almost always a spatially focal event. Indeed, mechanical testing of excised tissue samples following aortic surgery has demonstrated the patient-specific regional variability of aortic mechanical properties, even within the same lesion [4]. Beyond regionally heterogeneous material properties, the kinematics of the healthy or pathologic aortic wall may also be directly affected by asymmetric peri-aortic constraints from surrounding structures in vivo, particularly the vertebrae [5]. Thus, clinically relevant and accurate quantification of regional aortic mechanics must be performed in vivo and be capable of differentiating heterogeneities both circumferentially around and axially along the aorta. Unfortunately, quantifying circumferentially heterogeneous aortic wall strain has been a significant challenge due to the thinness of the wall (approximately 1–2 mm), relatively small motion during the cardiac cycle, and close approximation to neighboring structures. Indeed, the few prior attempts using feature-tracking in CT, speckle-tracking in 4D ultrasound, or velocity-mapping in phase contrast (PC) CMR have provided only simple indices of overall heterogeneity (as opposed to specific strain values) or strain over large regions (e.g., anterior vs. posterior walls) [6–8].

Recently, we presented a novel method of quantifying in vivo regional circumferential Green strain at discrete sectors around the aortic wall using 2D cine Displacement ENcoding with Stimulated Echoes (DENSE) CMR [9] – a technique capable of sub-voxel resolution of tissue kinematics by encoding the discrete 2D displacement of tissue in the phase data of each voxel. DENSE CMR was initially developed to evaluate myocardial strain [10, 11], and one initial study had shown its feasibility in assessing gross heterogeneities in stretch in the ascending aorta [12]. Using an optimized cine DENSE sequence and new post-processing techniques, our prior pilot study (*n* = 6) demonstrated the ability of DENSE to quantify 16 heterogeneous circumferential strain values around the aortic wall from a single 2D aortic cross-section [9]. Before attempting to map and clinically interpret the complex pathological strain distributions in various aortopathies, the current study aims to utilize this technique for the first time to explore the ‘baseline’ regional distributions of circumferential wall strain in non-aneurysmal aortas of varying ages (18–65 years, *n* = 30) at three clinically relevant locations along the descending thoracic and abdominal aorta: the distal aortic arch (site of initiation of Type B aortic dissection), the pericardiac descending thoracic aorta (site of propagation of thoracic aortic aneurysms and dissections), and the infrarenal abdominal aorta (site of abdominal aortic aneurysms).

## Methods

### Imaging

**Fig. 1:**
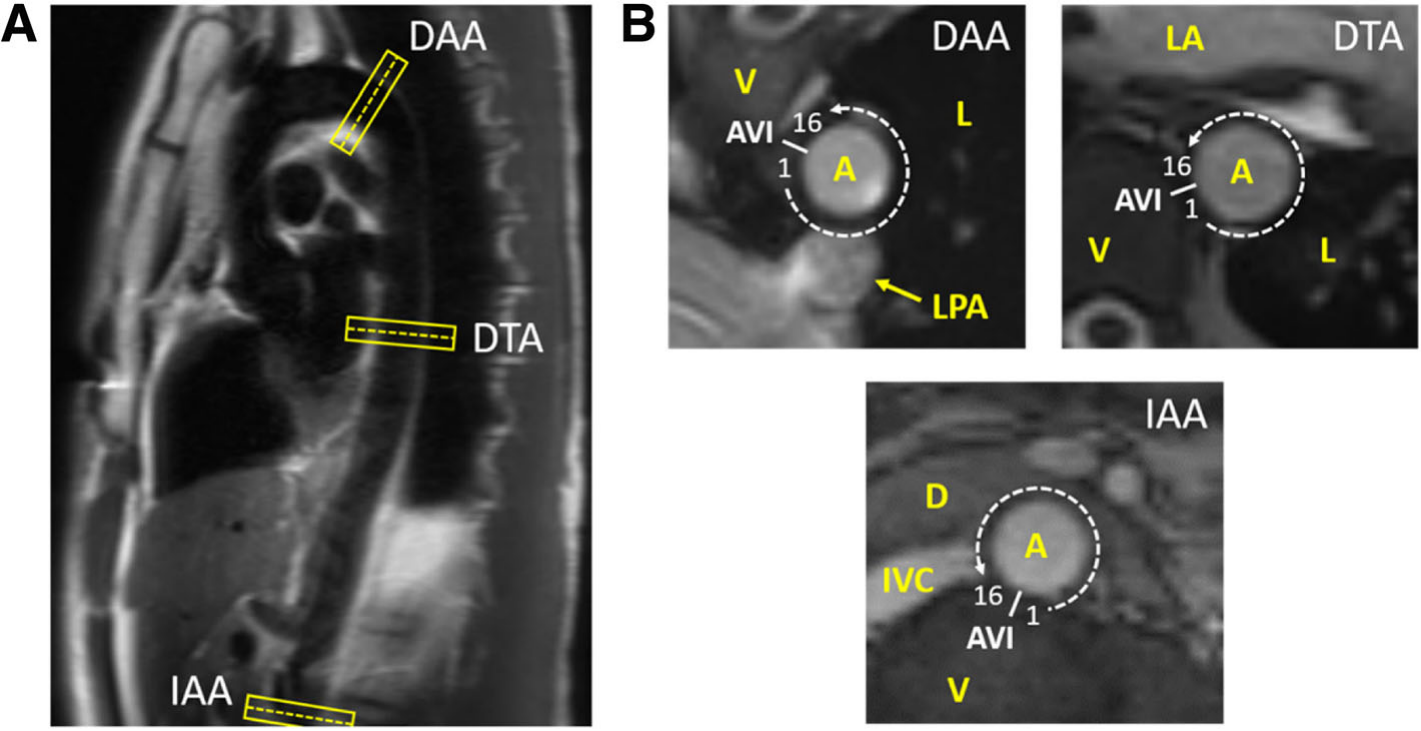
(**a**) Representative locations and orientations of the three axial cross-sections of interest on a sagittal-oblique HASTE image of the thoracoabdominal aorta. (**b**) Representative cross-sectional bSSFP images of the distal aortic arch (DAA), descending thoracic aorta (DTA), and infrarenal abdominal aorta (IAA) demonstrating local anatomy, location of the aorto-vertebral interface (AVI), and numbering of 16 sectors around the aortic wall. [HASTE – half-Fourier acquisition single-shot turbo spin-echo, bSSFP – balanced steady-state free procession, A – aorta, V – vertebra, L – lung, LPA – left pulmonary artery, LA – left atrium, D – duodenum, IVC – inferior vena cava]

Following approval by the Institutional Review Board at Emory University and optimization of the CMR protocol, 30 healthy adult subjects without history of aortopathy (excluding hypertension) provided written informed consent and underwent non-contrast CMR of the aorta at one or more axial locations on either a 3T Siemens Trio or Prisma scanner (Siemens Healthineers, Erlangen, Germany) with an 18-channel body matrix in combination with the table-mounted spine coil array. In total, scans were completed in 11 infrarenal abdominal aortas (IAA), 17 descending thoracic aortas (DTA), and 11 distal aortic arches (DAA). These data include six scans (two from each location) included in our previously published pilot study [9]. The IAA location was selected distal to the left renal artery near the level where the retroperitoneal fourth portion of the duodenum crosses the aorta. The DTA location was selected at the level of the left atrium near the mitral valve. The DAA location was selected just distal to the origin of the left subclavian artery (Fig. 1).

Following acquisition of survey images and sagittal-oblique half-Fourier acquisition single-shot turbo spin-echo (HASTE) imaging of the length of the thoracoabdominal aorta to aid in slice-selection, 2D cine balanced steady-state free procession (bSSFP) and spiral cine DENSE images were acquired normal to the local longitudinal axis of the aorta at the axial location of interest. bSSFP imaging was acquired with electrocardiographic (ECG) gating during a single breath-hold (voxel dimension 1.3 × 1.3 × 6 mm, TR 41.64 ms, TE 1.52 ms, flip angle 37^o^, GRAPPA acceleration factor 3), followed by navigator and cardiac gated 2D cine DENSE with spiral k-space sampling and fat suppression [11] (voxel dimension 1.3 × 1.3 × 8 mm, TR 16 ms, TE 1.21 ms, flip angle 15^o^, in-plane displacement encoding frequency *k*_*e*_ =0.17–0.25 cyc/mm, 18 spiral interleaves, 2 leaves per heartbeat, 4 signal averages). In order to capture the motion through local systole, 18 temporal sets of DENSE images were acquired every 32 ms following the ECG trigger.

### Post-processing and calculation of strain

Post-processing and analysis was performed offline using custom code in MATLAB (MathWorks, Natick, Massachusetts, USA) as detailed in Wilson et al. [9]. Briefly, after unwrapping the DENSE phase images and manually segmenting the luminal and adventitial boundaries of the aortic wall at each time point using the DENSE magnitude images, the components of the displacement vector ***d***_*t*_ (relative to the reference configuration at the time of encoding immediately following the ECG trigger) for each voxel in the wall at time *t*∈ [1,18] were calculated according to:

1 $$ {d}_{i,t}=\kern0.5em {\varphi}_{i,t}/2\pi {k}_e,\kern0.5em i=x,y, $$

where *d*_*i*,*t*_ is the component in the *i*-direction at time *t*, and *φ*_*i*,*t*_ is the unwrapped *i*-phase imaging value for the voxel at time *t*. Similar to previous reports [13], the forward displacement vector (***u***_*t*_) and tracked position (***X***_*t*_) of each voxel in the reference configuration was then calculated through all 18 timepoints via interpolation using the three closest back-projected displacement vectors (***d***_*t*_) at each time *t*. To reduce the noise in the displacement data secondary to imaging the thin moving aortic wall, algorithms for time-smoothing the position data and spatially smoothing the displacement vector data were utilized as previously described [9], with the exception that the minimum amount of displacement vector smoothing for IAAs was increased from averaging all data within one voxel-space of the voxel of interest to two voxel-spaces.

The 2D Green strain (***E***) was then calculated over 16 equally spaced sectors around the circumference of the aortic wall using weighted spatial averaging and reference point averaging as a function of the referential displacement gradient, $\boldsymbol{H}=\kern0.5em \frac{\partial \boldsymbol{u}}{\partial \boldsymbol{x}},$ by

2 $$ \boldsymbol{E}=\frac{1}{2}\left(\boldsymbol{H}+{\boldsymbol{H}}^T+{\boldsymbol{H}}^T\cdot \boldsymbol{H}\right), $$

via a quadrilateral finite element method with normalized coordinates as described by Humphrey [14] and detailed in Wilson et al. [9]. Finally, the results were transformed into radial-circumferential coordinates using a rotation defined by the local curvature of the aortic wall at the point of interest and time-smoothed with a fifth-order polynomial. For this study, ‘peak’ systolic circumferential strain distributions were calculated at the timepoint with the largest mean circumferential strain for the entire cross-section in the IAA and DTA, or at the mean timepoint of maximum circumferential strain of the first six sectors that peak following the beginning of local systole in the DAA (due to some sectors peaking in local diastole in select subjects).

For geometric comparisons between subjects, diastolic aortic diameter was calculated as the average of the manually assessed aortic diameter in the *x* and *y* directions of the imaging plane (generally the right lateral-left lateral and anterior-posterior dimensions, respectively, except in the DAA due to its oblique orientation). A relative aortic size was also calculated as the aortic diameter divided by the body surface area (BSA) [15, 16]. For comparison of strain distributions between subjects, normalized circumferential strain (NCS) was calculated by dividing the circumferential strain of the sector of interest by the mean circumferential strain of that subject at the time of peak systolic circumferential strain (i.e., local systole). Herein, note that maximum NCS refers to the highest value of NCS at any time point (not necessarily at the time of local systole). To account for slight differences in local anatomy between subjects, numbering of the 16-sector strain map of each subject begins at the first sector counterclockwise to the nearest apposition of the aorta and vertebral column at the given cross-section (i.e., the aorto-vertebral interface (AVI) (Fig. 1). Note that due to the patient-specific oblique cross-sections required at the DAA, the AVI at this location was defined from the anterior tip of the nearest in-plane vertebra. To further improve the comparison of strain maps between patients in the oblique DAA cross-sections (which were non-parallel due to patient-specific angles of curvature of the aortic arch), each 16-sector map was allowed a ± 1 sector rotation to align the sectors with maximum/minimum circumferential strain at local systole. Finally, for interpatient comparison of the degree of regional heterogeneity of the strain distribution, a heterogeneity index [17] was calculated as the standard deviation of the 16 regional circumferential strain measurements at local systole divided by the mean circumferential strain of the entire cross-section.

### Reproducibility

Intra-observer and inter-observer reproducibility of regional NCS was evaluated in 6 scans (two at each location, c.f. [9]) using the coefficient of variation (CoV) of the sector differences and mean absolute values of difference. Reproducibility was assessed when comparing all 16 circumferential sectors per scan, as well as when comparing only the two local NCS maxima and two local NCS minima sectors per scan. Intra-observer data was calculated from Reader 1 re-processing the DENSE images at least 6 months after the first segmentation. Inter-observer data was calculated by comparing the difference between the experienced Reader 1 and an independent Reader 2, who was recently trained to process aortic DENSE. Inter-observer values are reported as the mean of the differences between Reader 2 and each analysis by Reader 1. The coefficient of variation (CoV) was calculated as the standard deviation of the non-absolute difference of the NCS values divided by the mean NCS value (which equals 1 in all cases due to the normalization). CoV values were qualitatively rated as good (≤0.20), fair (0.21–0.30), or poor (> 0.30).

### Statistics

Statistical comparisons were performed on SPSS (Ver. 26, Statistical Package for the Social Sciences, International Business Machines, Inc., Armonk, New York, USA) and Excel (2016, Microsoft, Redmond, Washington, USA). Comparisons of mean values of key demographic and strain metrics between various subgroups of subjects at each aortic location were conducted using Mann-Whitney U tests (significance, *p* < 0.05). Pearson correlation coefficients (ρ) were used to quantify the correlations of key strain metrics and age, with moderate correlation defined as |ρ| ≥ 0.5 and strong correlation as |ρ| ≥ 0.9. Differences between aortic locations related to the number of successful scans, sex distribution, and presence of medical comorbidities were evaluated using Fisher’s exact test (significance, *p* < 0.05). Finally, Kruskal-Wallis tests with Bonferroni correction were used to compare differences in mean values of age, body mass index (BMI), body surface area (BSA), aortic diameter, relative aortic diameter, mean circumferential strain at local systole, maximum NCS, and heterogeneity index between aortic locations (significance, p < 0.05 after correction).

## Results

Complete analyzable results were achieved in 10/11 IAA, 13/17 DTA, and 9/11 DAA scans, for a success rate of 82%. Five subjects had complete scans at more than one location (4 DTA/DAA, 1 DTA/IAA). One IAA scan was eliminated due to an accessory left renal artery at the cross-section of interest. Four DTAs were eliminated due to patient motion (1), poor signal (2), and physical deformation of the aorta at the reference configuration by the heart (1). Two DAAs were eliminated due to poor signal (1) and inadequate encoding frequency preventing accurate phase-unwrapping (1). Notably, the final 7 scans at each location were successful, suggesting an improvement in technique with increased experience. Demographics and self-reported medical history of the subjects are shown in Table 1.

**Table 1:** Demographics and self-reported medical history of subjects. [S.D. – standard deviation, BMI – body mass index, BSA – body surface area, AAD – aortic aneurysm and/or dissection, *one subject did not report, ^three subjects did not report]

	IAA	DTA	DAA	*p*	*p* (IAA-DTA)	*p* (IAA-DAA)	*p* (DTA-DAA)
Successful Scans (Attempts)	10 (11)	13 (17)	9 (11)	0.87	–	–	–
Sex (F / M)	4 / 6	5 / 8	5 / 4	0.74	–	–	–
Age (± SD)	36 ± 14	37 ± 15	41 ± 16	0.81	–	–	–
BMI (kg/m^2^)	23 ± 3	27 ± 5	24 ± 10*	0.08	–	–	–
BSA (m^2^)	1.8 ± 0.2	2.0 ± 0.2	1.9 ± 0.3*	0.29	–	–	–
Aortic Diameter (cm)	1.6 ± 0.2	2.1 ± 0.3	2.3 ± 0.3	< 0.001	0.004	< 0.001	0.46
Rel. Aortic Size (cm/m2)	0.9 ± 0.1	1.1 ± 0.1	1.2 ± 0.1*	< 0.001	0.054	< 0.001	0.16
HTN	1	3	3	0.42	–	–	–
Diabetes	0	1	0	1.00	–	–	–
Renal Dysfunction	0	0	0	1.00	–	–	–
Dyslipidemia	0	1	1	0.74	–	–	–
Family History of AAD	1	1^	1*	1.00	–	–	–
Current Smoker	0	1	1	0.74	–	–	–
Prior Smoker	0	3	3	0.14	–	–	–

### Infrarenal abdominal aorta (IAA)

**Fig. 2:**
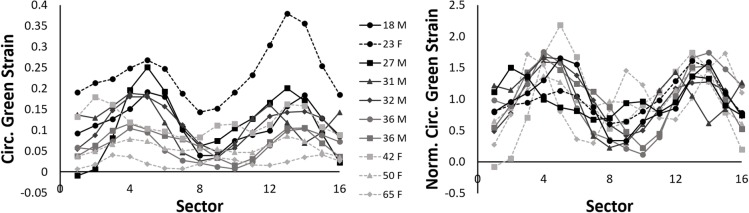
(Left) Circumferential Green strain at local systole at 16 sectors equally spaced around the IAA wall in 10 subjects of various ages. (Right) Normalization of the circumferential strain by the patient-specific mean circumferential strain at local systole across the entire cross-section

Figure 2 demonstrates the interpatient variability in the spatial distribution of circumferential Green strain at peak local systole in 10 IAAs of various ages before and after normalization. Note the expected trend of decreasing magnitudes of strain as age increases for both males and females on average, but the general similarity of normalized strain distribution in terms of focal regions of high and low strain.

**Fig. 3:**
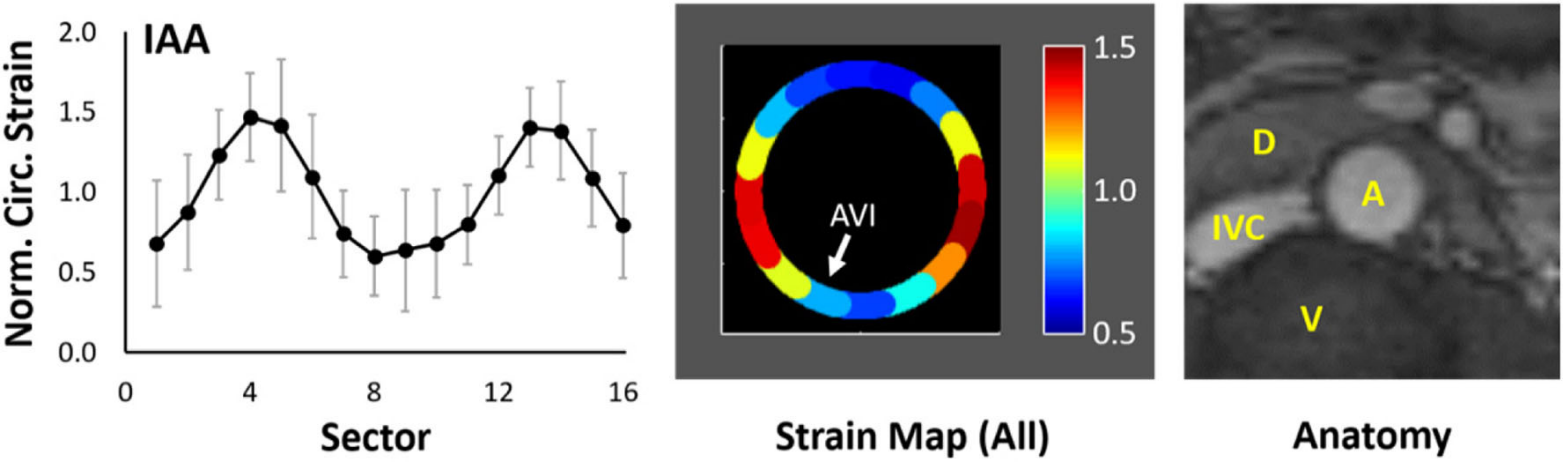
(*Left*) Plot of mean regional normalized circumferential strain (NCS) (±S.D.) at local systole of 10 IAAs. (*Center*) Strain map of mean regional NCS oriented to local anatomy. White arrow identifies the location of the aorto-vertebral interface (AVI). (*Right*) Representative local anatomy at the cross-section of the IAA for reference. [S.D. – standard deviation, A – aorta, V – vertebra, IVC – inferior vena cava, D – fourth portion of duodenum]

After combining data over all subjects, Fig. 3 demonstrates the mean distribution of NCS in the IAA and displays the resulting average strain map relative to the local anatomy. Note the highest strains in the lateral walls (peaking at 1.47 ± 0.27 in Sector 4), with lower strains along the anterior and posterior walls that abut the duodenum/retroperitoneal fascia and vertebra, respectively. Comparing males (*n* = 6) to females (*n* = 4) revealed a nonsignificant difference in age (30 ± 7 vs. 45 ± 18 years, *p* = 0.17), mean circumferential strain (0.10 ± 0.03 vs. 0.11 ± 0.09, *p* = 1.00), and heterogeneity index (0.49 ± 0.10 vs. 0.32 ± 0.11, *p* = 0.11), but a significant difference in the location of maximum NCS (3.3 ± 2.7 vs. -1.3 ± 2.4 sectors counterclockwise from the AV interface, *p* = 0.04). Aortic diameter, relative aortic size, body mass index (BMI), and BSA were not significantly different.

### Descending thoracic aorta (DTA)

**Fig. 4:**
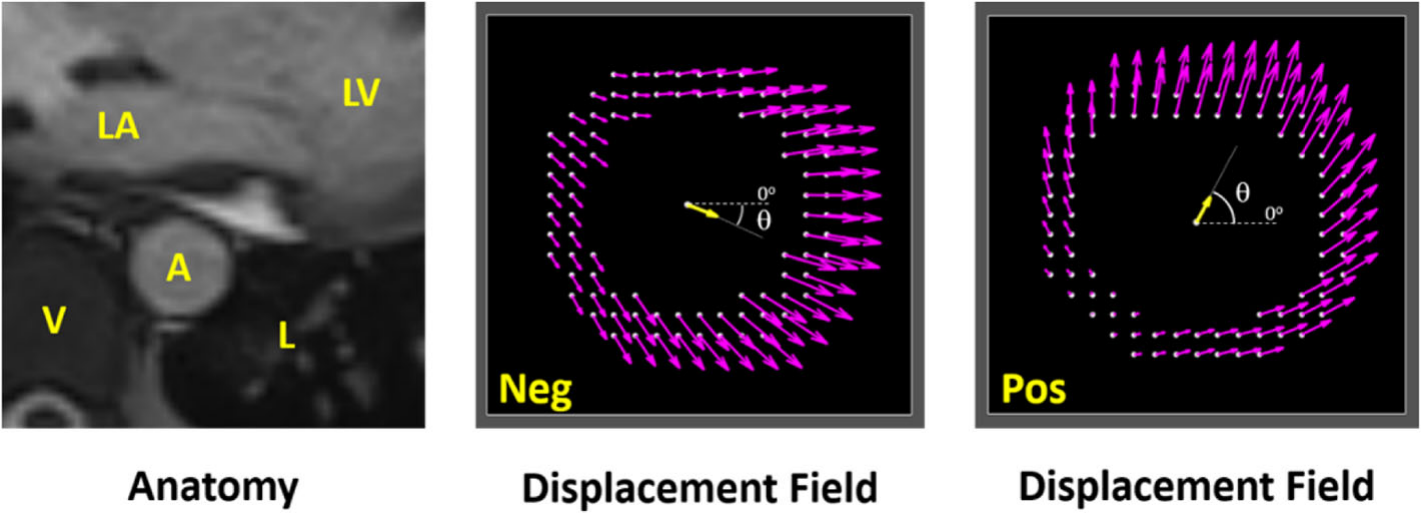
(*Left*) Representative local anatomy at the cross-section of the DTA. [A – aorta, V – vertebra, L – lung, LA – left atrium, LV – left ventricle] (*Center*) Representative peak displacement field of a negatively displacing DTA. Yellow arrow represents the mean displacement; θ represents the mean displacement angle. (*Right*) Representative peak displacement field of a positively displacing DTA

Scans of the DTA demonstrated greater interpatient variability of strain distribution than at the IAA. Due to clear differences in the mean displacement of the aorta, scans of the DTA were first divided into two subgroups based on the sign of the mean displacement angle at peak local systole relative to a zero angle pointing in the left lateral direction in standard transverse imaging (Fig. 4).

**Fig. 5:**
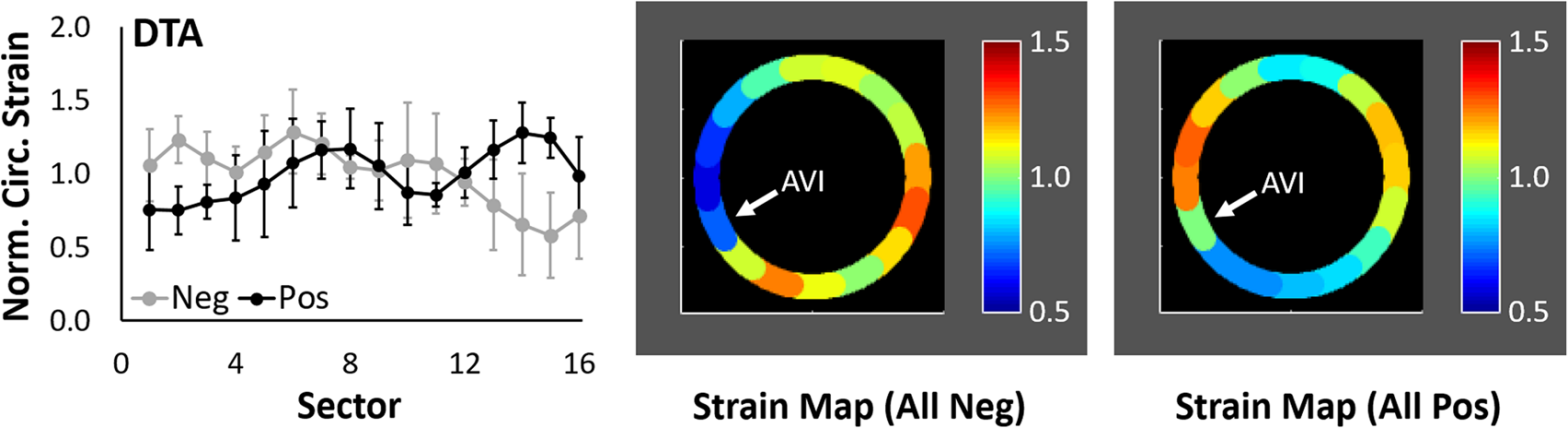
Plots of mean regional NCS (±S.D.) at local systole and corresponding strain maps of 4 DTAs with negative mean displacement and 9 DTAs with positive mean displacement. White arrow identifies the location of the aorto-vertebral interface (AVI)

Nine subjects had positive angles (anterior displacement) and four had negative angles (posterior displacement) (67 ± 23 vs. -24 ± 3^o^, *p* = 0.003). The mean distribution of NCS at local systole and the corresponding strain map for each group are shown in Fig. 5. Peak NCS at local systole was 1.28 ± 0.20 in Sector 14 (medial wall) in the positive group and 1.29 ± 0.29 in Sector 6 (lateral wall) in the negative group. The positive group was significantly older than the negative group (43 ± 15 vs. 24 ± 1 years, *p* = 0.03), with 4 of 5 patients younger than 30 years being in the negative group. Similarly, aortic diameter was greater in the positive group (2.2 ± 0.3 vs. 1.8 ± 0.1 cm, *p* = 0.006); however, the relative aortic size, BMI, and BSA were not significantly different. Differences in mean circumferential strain and maximum circumferential strain between the positive and negative groups neared significance (0.11 ± 0.04 vs. 0.16 ± 0.04, *p* = 0.11; 0.17 ± 0.06 vs. 0.24 ± 0.07, p = 0.11), but no significant differences were noted in maximum NCS or heterogeneity index (1.47 ± 0.11 vs. 1.53 ± 0.22, *p* = 0.83; 0.28 ± 0.07 vs. 0.29 ± 0.07, *p* = 1.00). All patients with a history of hypertension (*n* = 3) were in the positive group. No significant differences in metrics were noted when comparing males (*n* = 8) to females (*n* = 5) in this small exploratory study.

### Distal aortic arch (DAA)

**Fig. 6:**
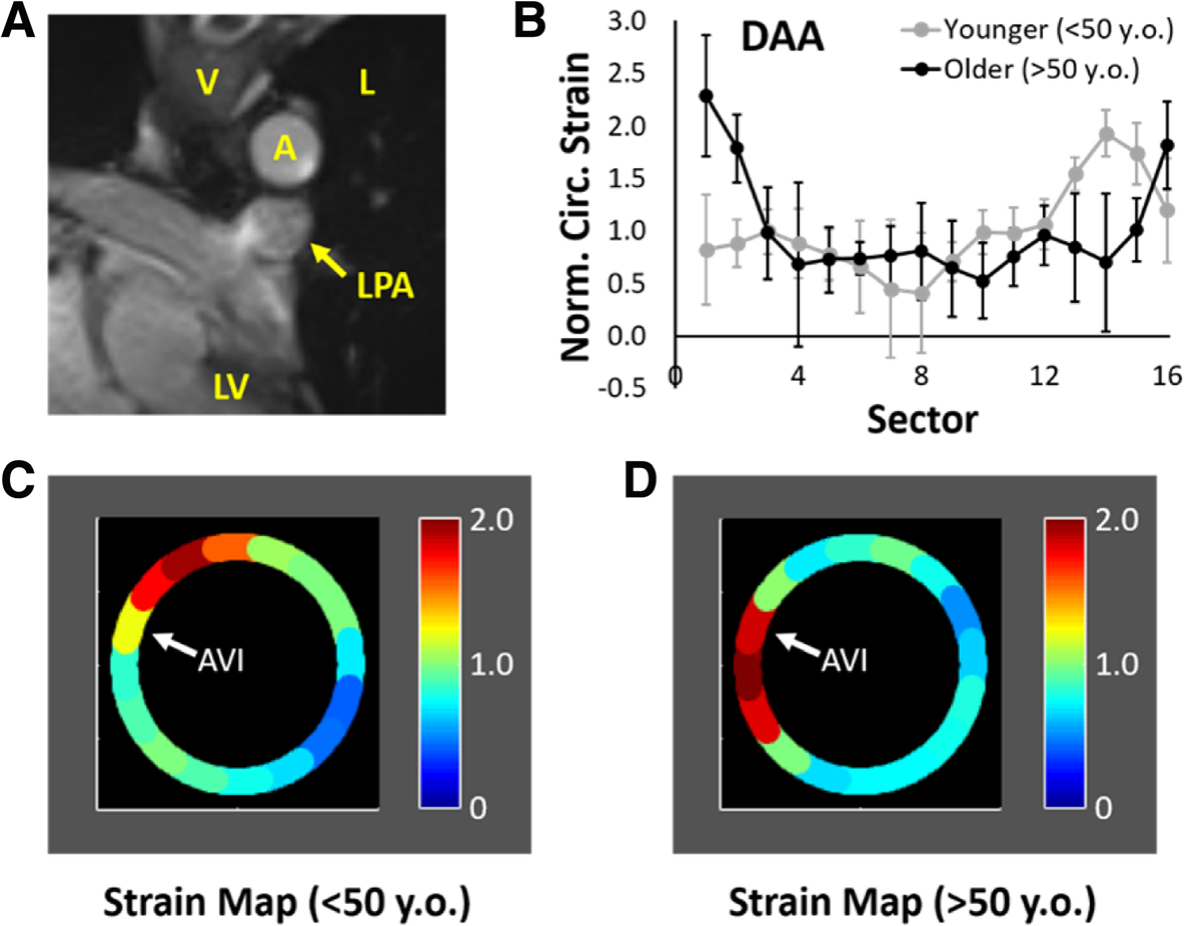
(**a**) Representative local anatomy at the oblique cross-section of the DAA. [A – aorta, V – vertebra, L – lung, LPA – left pulmonary artery, LV – left ventricle].(**b**-**d**) Plots of mean regional NCS (±S.D.) at local systole and corresponding strain maps of 5 DAAs from subjects < 50 years and 4 DAAs from subjects > 50 years-old. White arrow identifies the location of the aorto-vertebral interface (AVI)

Results of the DAA scans fell into two primary strain distributions that correlated with age (Fig. 6). Relative to the local anatomy, peak NCS at local systole occurred in the superior curvature on average in subjects < 50 years (1.93 ± 0.22 in Sector 14) but in the medial wall in subjects ≥50 years (2.29 ± 0.58 in Sector 1). Comparing the younger group (29 ± 9 years) with the older group (57 ± 3 years) revealed significant differences in the location of maximum NCS relative to the AVI (− 2.4 ± 0.5 vs. 1.5 ± 1.3 sectors counterclockwise from the AVI, *p* = 0.02) and in aortic diameter (2.5 ± 0.3 vs. 2.1 ± 0.2 cm, *p* = 0.03), but no significant difference in maximum circumferential strain (0.22 ± 0.08 vs. 0.15 ± 0.05, *p* = 0.29), mean displacement angle at local systole (19 ± 6^o^ vs. 46 ± 34^o^, *p* < 0.19), or heterogeneity index (0.53 ± 0.14 vs. 0.62 ± 0.20, *p* = 0.73). Differences in mean circumferential strain at local systole (0.11 ± 0.04 vs. 0.05 ± 0.03, *p* = 0.06) and maximum NCS (2.0 ± 0.2 vs. 3.1 ± 0.9, p = 0.06) neared significance. There were no significant differences in relative aortic size, BMI, or BSA.

Three patients with scans of the DAA reported a history of hypertension, and all were in the older group. Interestingly, the timing of maximum NCS at any sector relative to the timing of local peak circumferential strain was significantly different in hypertensive subjects compared to non-hypertensive subjects (4.7 ± 2.1 vs. 0.5 ± 1.4 timepoints after local systole, *p* = 0.02). All three hypertensive subjects experienced maximum circumferential strain at least two timepoints after local systole, while only 1 of 6 non-hypertensive patient did. The largest two values of maximum NCS (3.3 and 4.1) came from two of the hypertensive subjects. In comparison of males (*n* = 4) to females (*n* = 5), the difference in mean age was not significant (36 ± 16 vs. 45 ± 17, *p* = 0.73), though 3 of 4 of the older group were female, but the average maximum NCS (1.88 ± 0.11 vs. 2.95 ± 0.81, p = 0.02) and heterogeneity index (0.41 ± 0.06 vs. 0.69 ± 0.09, p = 0.02) were both significantly greater in females in this small exploratory study.

### Correlations between metrics and locations

**Fig. 7:**
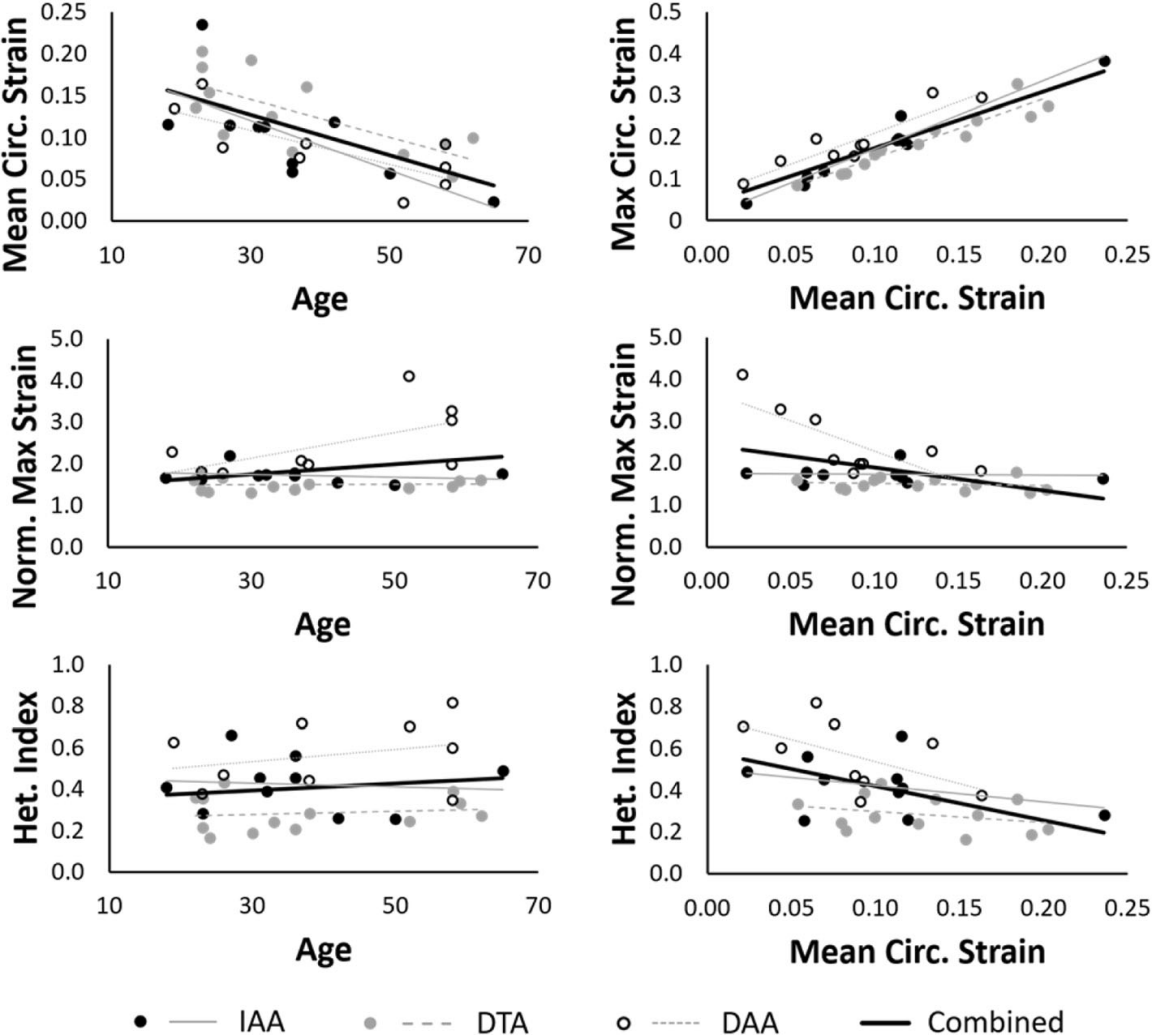
Graphical correlations of age, mean circumferential strain at local systole, maximum normalized circumferential strain (NCS), and heterogeneity index for each aortic location and for the combined data set

Pearson’s correlations between key biomechanical metrics of strain within each group are presented in Table 2 and graphically highlighted in Fig. 7, along with correlations when evaluating the data at all locations as a whole. Due to small sample sizes at each location, statistical significance is recorded only for the combined data. Comparison of BMI, BSA, aortic diameter, and relative aortic size to age, mean strain, maximum NCS, and heterogeneity index revealed no absolute value of Pearson’s correlation ≥0.5 when evaluating the data set as a whole.

**Table 2:** Select values of Pearson’s correlation (ρ) between age, mean circumferential strain at local systole, maximum circumferential strain, maximum normalized circumferential strain (NCS), and heterogeneity index at each aortic location and for the combined data set. [*p < 0.05, **p < 0.01, ***p < 0.001]

Aortic	Age vs.	Age vs.	Age vs.	Age vs.	Mean Strain vs.	Mean Strain vs.	Mean Strain vs.
Location	Mean Strain	Max Strain	Max NCS	Het. Index	Max Strain	Max NCS	Het. Index
IAA	−0.71	−0.75	−0.22	−0.09	0.98	−0.06	−0.34
DTA	−0.71	−0.71	0.04	0.13	0.95	−0.16	− 0.31
DAA	−0.75	− 0.63	0.61	0.28	0.92	−0.77	−0.55
Combined	−0.69***	− 0.67***	**0.30**	**0.15**	0.90***	−0.46**	− 0.50**

**Fig. 8:**
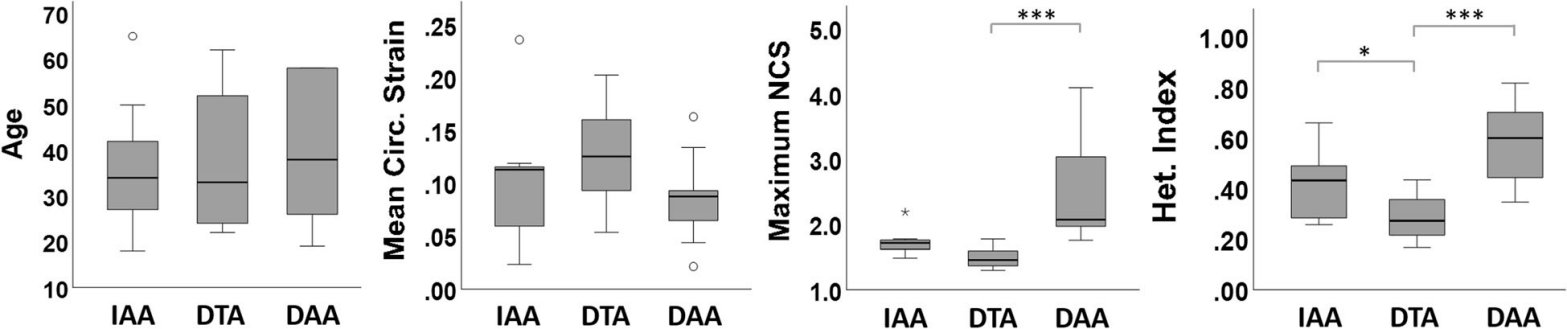
Box and whisker plots and statistical comparison of the age, mean circumferential strain at local systole, maximum NCS, and heterogeneity index at each aortic location. [*p < 0.05, **p < 0.01, ***p < 0.001]

Although this study was not specifically designed to compare average metrics of strain between different aortic locations (IAA, DTA, DAA), basic statistical analysis using Kruskal-Wallis with Bonferroni correction indicated significant differences in maximum NCS between DTA/DAA (*p* < 0.001) and in heterogeneity index between IAA/DTA (*p* < 0.05) and DTA/DAA (p < 0.001), as shown in Fig. 8. The difference in maximum NCS between IAA/DTA and IAA/DAA approached significance (*p* = 0.08 and *p* = 0.07, respectively). Consistent with the known tapering of the aorta from its origin to the iliac bifurcation, the mean aortic diameter and relative aortic size decreased from the DAA to the IAA (Table 1), although significant differences in aortic diameter were only recorded between IAA/DTA and IAA/DAA. Neither age (*p* = 0.81), mean circumferential strain at local systole (*p* = 0.13), nor maximum circumferential strain (*p* = 0.88) were significantly different between aortic locations in this small population that included a wide range of ages. The notable outlier observed in the IAA group for mean circumferential strain was a 23 year-old female with a prominent left renal vein overlying the anterior wall of the aorta at the location of the scan. The outlier in the IAA group for maximum NCS was a healthy 27 year-old male.

### Reproducibility

Mean metrics quantifying intra-observer and inter-observer reproducibility of regional NCS from six subjects are summarized in Table 3 by aortic location and for the data set as a whole. The absolute mean difference evaluates all 16 regional sector values from each scan; the absolute local extrema mean difference considers only the two primary local NCS maxima and two primary local NCS minima sectors per scan. The absolute local extrema sector difference represents the absolute mean difference in sector number (i.e., circumferential location) for each of the local extrema. The coefficient of variation (CoV) represents the standard deviation of the non-absolute difference of the NCS values divided by the mean NCS value.

**Table 3:** Reproducibility metrics of normalized circumferential strain (NCS) from the analysis of six subjects by comparing results from the same reader processing the data at least six months apart (intra-observer) and by comparing results from two independent readers (inter-observer). [Abs. – absolute value, SD – standard deviation, CoV – coefficient of variation, Local Ext. – Local max/min extrema]

	Aortic	Abs. Mean	CoV	Abs. Max/Min	CoV	Abs Max/Min
	Location	Diff. (±SD)	(Mean Diff.)	Diff. (±SD)	(Max/Min)	Sector Diff.
	IAA	0.09 ± 0.06	0.11	0.07 ± 0.04	0.08	0.4
Intra-	DTA	0.15 ± 0.13	0.20	0.11 ± 0.07	0.13	0.8
Observer	DAA	0.21 ± 0.15	0.27	0.14 ± 0.17	0.20	0.3
	Mean	**0.15 ± 0.11**	**0.19**	**0.10 ± 0.09**	**0.14**	**0.5**
	IAA	0.12 ± 0.08	0.15	0.11 ± 0.09	0.15	0.6
Inter-	DTA	0.13 ± 0.09	0.17	0.12 ± 0.09	0.15	0.3
Observer	DAA	0.32 ± 0.21	0.39	0.31 ± 0.23	0.32	0.3
	Mean	**0.19 ± 0.13**	**0.24**	**0.18 ± 0.13**	**0.21**	**0.4**

## Discussion

The importance of vascular mechanics to the health and pathological remodeling of the aorta has been increasingly recognized, particularly with regards to the correlation of patient-specific variation and clinical risk. As a result, there is a clear need for a reliable non-invasive technique for quantifying patient-specific metrics of regional aortic mechanical function. Herein, a recently developed novel application of cine DENSE CMR was used to systematically map the heterogeneous kinematics of non-enlarged aortas at three axial locations prone to aortic aneurysms and dissections to explore normal baseline distributions of regional in vivo circumferential strain throughout adulthood for the first time.

In brief, the results demonstrate that normal aortic kinematics are regionally heterogeneous around the circumference of the aorta at each location evaluated in this study. However, strain distributions can be successfully grouped into similar patterns following normalization and proper group assignment based on a combination of aortic location, age, mean displacement, and potentially sex or history of hypertension, though larger dedicated studies are required to verify the independent roles of these co-factors. Thus, patient-specific strain mapping and its comparison to carefully selected control data may provide new and potentially useful information for direct correlation with clinical metrics (e.g., specific diagnoses, risk stratification, or clinical outcomes). The results also suggest that bulk aortic motion and patient-specific peri-aortic constraints secondary to surrounding structures may strongly influence the unique distributions of strain at each location. Thus, while quantifying regional kinematics may provide valuable information for improving patient-specific models of aortic mechanics, computational engineers must carefully consider that these heterogeneous kinematics may reflect both regionally heterogeneous material properties and regionally heterogeneous mechanical boundary conditions. The following discussion further evaluates some of the unique findings at each aortic location and their potential clinical significance, as well as discussion of general correlations and limitations of the study.

### Infrarenal abdominal aorta (IAA)

Following normalization, mapping of peak circumferential strain in the IAA demonstrates a consistent heterogeneous distribution of strain with focally higher strains in the lateral walls compared to the anterior and posterior walls (Fig. 3). This distribution in non-dilated aortas is notable given that the location of abdominal aortic aneurysm rupture favors the right or left lateral walls compared to the anterior or posterior wall (65% lateral, 17.5% anterior, 17.5% posterior [18]). Low strain and low displacement in the posterior wall were anticipated, consistent with tethering of the aorta to the neighboring vertebra. Due to this constraint, the mean displacement of the IAA during systole is anterior, consistent with previous reports [19]. As a result, the higher strain in the lateral walls may reflect a strain concentration as the aorta pulls away from its fixation to the vertebra during systole. This effect emphasizes the importance of considering boundary conditions in interpreting kinematic heterogeneities and not just assuming all heterogeneities relate solely to regional differences in material properties of the wall itself. Interestingly, the anterior wall also demonstrated low strain, but with high displacement, suggesting bulk motion. At the location of the scan, the anterior wall abuts the retroperitoneal fascia and/or the fourth portion of the duodenum. These structures may provide external support to the anterior aorta but are not immovable like the vertebra. However, inherent asymmetric stiffening of the anterior aortic wall in adulthood due to persistent asymmetric bloodflow [20] and/or cyclic stretch may also contribute. Future evaluation in pediatric subjects and ex vivo mechanical testing of tissue samples would be beneficial for further evaluation.

With regards to sex differences, the observed significant difference in the site of maximum NCS between men and women (5 of 6 in the left lateral wall for men and 3 of 4 in the right lateral wall for women) is unexpected. To our knowledge, no preferential lateralization of IAA wall properties or failures have been reported between sexes. This correlation should be followed in future studies with larger numbers of participants.

### Descending thoracic aorta (DTA)

In general, results from the DTA suggest a change in mean displacement of the aorta in adulthood at approximately 30 years of age. The mean displacement of the DTA toward the left posterior lung space in younger adults may be due to either greater compliance in the tethering/mediastinal support of the aorta in younger adults or potentially cardiac effects that were not quantified in this study (e.g., heart rate, timing of left atrial filling and contraction, etc.). Notably, posteriorly (negatively) displacing aortas concentrate circumferential strain counterclockwise from the AV interface while anteriorly (positively) displacing aortas concentrate strain clockwise (Fig. 5a), consistent with the aorta pulling away from a fixation to the vertebra in the direction of mean displacement.

The older adults generally demonstrated a mean positive displacement toward the left ventricle at local systole and a bimodal distribution of peak circumferential strain. Elevated strain occurred along the medial wall next to the esophagus and the left lateral wall adjacent to the lung, with reduced strain anteriorly and posteriorly. Interestingly, this distribution coincides with a common arrangement of type B aortic dissections that extend distally into the mid-DTA where the free walls of the true lumen and false lumen lie medially and laterally, respectively, and the dissection flap inserts anteriorly and posteriorly to the wall. Future studies will be required to investigate the potential for focal regions of low cyclic strain to resist the circumferential propagation of dissection around the aorta.

### Distal aortic arch (DAA)

Strain distributions in the DAA demonstrated the clearest differentiation between subjects grouped by age. Those < 50 years (younger group) demonstrated peak circumferential strain at local systole in the superior curvature (Fig. 6), as would be expected due to the asymmetric flow at this location where blood is redirected inferiorly into the DTA. In subjects ≥50 years (older group), the location of peak strain significantly shifted toward the medial/inferomedial wall between the AV interface and the apposition of the aorta and left pulmonary artery (Fig. 6). Notably, the ligamentum arteriosum connects these two structures near this apposition. In all three subjects with hypertension (all > 50 years), but only 1 of 6 non-hypertensive subjects, the maximum NCS occurs in early local diastole (> 2 timepoints after local peak systole) in this same region. Two potential mechanisms may contribute to this observation. First, the persistent asymmetric blood flow along the superior curvature may lead to selective stiffening of this region, resulting in a relative increase in NCS in the inferior curvature over time. Secondly, increasing age and hypertension may increase the general stiffness of the ascending aorta, aortic arch, and mediastinal connective tissue, allowing the ligamentum arteriosum to transmit the inferior displacement of the heart and pulmonary trunk during late cardiac systole to the inferior wall of the aortic arch. As a result, tension is increased in the inferomedial wall of the inferior curvature between the fixation at the AV interface and the connection of the ligamentum arteriosum.

These results suggest that there are two potential locations of elevated circumferential strain in the DAA: the superior curvature in younger/compliant aortas and the inferomedial wall in older/stiffer aortas. Furthermore, maximum circumferential strains that occur during local systole may favor the superior curvature, while maximum strain occurring in early local diastole may favor the inferior curvature. Notably, the entry tears of type B aortic dissections that occur in the DAA also are typically grouped by their distribution in either the superior curvature or inferior curvature [21, 22]; however, to our knowledge, there has been no data to date that directly correlates the site of entry tear to age, hypertension, or timing of maximum regional circumferential strain.

Compared to other regions of the aorta, the circumferential strain distribution of the DAA shared some similarities but a few notable differences. Similar to the DTA and IAA, regions with elevated strain often occurred within 1–2 sectors of the AVI – suggesting a potential strain concentration as the aorta pulled against a focal constraint during cyclic loading. However, the peak NCS tended to be higher in the DAA and occurred very close to the AV interface. This may reflect a less broad fixation to the vertebra at this region of the curved DAA, where the aorta may not be in full apposition to the vertebra as in the straight segments of the DTA and IAA. In addition, the difference in strain distribution of the DAA due to age was not significantly correlated to the average peak displacement angle as in the DTA. This may be secondary to the small number of samples, but could also suggest that the differences in strain distribution in the DAA with age may result from remodeling-induced material differences in the aortic wall or support structures over time and not just due to changes in bulk motion of the aorta.

### General correlations

Regarding correlations between age and metrics of strain (Table 2, Fig. 7), all aortic locations demonstrated a moderate negative correlation between age and both mean and maximum circumferential strain, as expected due to the general stiffening of the aorta over time. A strong positive correlation (ρ > 0.9) was also observed between mean and maximum circumferential strain, which could be useful for estimating maximum circumferential strain from standard cine CMR or CT images which are only capable of quantifying a single homogenized circumferential strain value per cross-section. Normalization of the maximum circumferential strain effectively eliminated these correlations in the IAA and DTA, demonstrating the usefulness of normalization to provide a comparison of strain distribution between patients with baseline differences in overall compliance. On the other hand, high values of maximum NCS in the oldest and stiffest DAAs revealed a persistence of a moderate correlation between maximum NCS and both age and mean circumferential strain at this location, highlighting the disproportionate increase in the relative maximum strain as the DAA ages/stiffens compared to the DTA and IAA. Indeed, the average maximum NCS was significantly greater in the DAA than the DTA (*p* < 0.001) and approached significance when compared to the IAA (*p* = 0.07) (Fig. 8). In addition, the DAA demonstrated significantly greater values of heterogeneity index than the DTA (*p* < 0.001). Whether these differences in normalized strain and regional heterogeneity relate to the much greater occurrence of aortic dissection initiating in the DAA than in the mid-DTA or IAA is unknown and should be further investigated in studies specifically powered to compare different aortic locations.

The heterogeneity index, which is a normalized metric of the variation of circumferential strain around the aorta, was not significantly correlated with age or mean circumferential strain, except for a moderate negative correlation in the DAA between heterogeneity index and mean strain. The clinical relevance of the heterogeneity index (and maximum NCS) being significantly greater in females in the DAA requires follow-up in a larger study dedicated to sex differences. Finally, the magnitude of correlation of both the heterogeneity index and the maximum circumferential strain was greater when compared to the mean circumferential strain than to age in all aortic locations, suggesting that a functional biological age as defined by mean strain better correlates to advanced strain metrics than simple chronological age.

### Reproducibility

The coefficients of variation in Table 3 suggest overall good intra-observer reproducibility (CoV = 0.19) and fair inter-observer reproducibility (CoV = 0.24). However, consideration of the different aortic locations suggests that the IAA and DTA have both good intra-observer and inter-observer reproducibility, while the DAA has fair intra-observer and poor inter-observer reproducibility. Notably, unlike the straight IAA and DTA, the DAA is often curved, which increases the challenge of a consistent manual segmentation, particularly when using a thick imaging slab (8 mm). Interestingly, considering only the local maxima and minima of the regional NCS values improves the mean reproducibility for all locations by neglecting slight shifts in the distribution of NCS (e.g., when differences in segmentation can lead to a local extrema being shifted by one sector clockwise or counterclockwise). Thus, the strict sector-to-sector reproducibility is affected not simply by the values of strain, but also by slight segmentation-dependent shifting in the sector maps. Notably, the overall mean difference in the circumferential locations of the local extrema is ≤0.5 sectors (an angle of approximately 11^o^), suggesting the general distributions of strain are consistent within and between observers. For this exploratory study, identifying these unique spatial distributions (e.g., maximum NCS occurring in the superior curvature of younger DAAs but in the medial/inferomedial wall in older DAAs, Fig. 6) was a main finding independent of exact magnitudes of the strain. Efforts are currently underway to automate/semi-automate the segmentation process which should significantly improve both intra-observer and inter-observer reproducibility.

### Limitations

A few challenges and limitations of this approach are worth elucidating. First, achieving adequate signal and reducing noise is critical in aortic DENSE CMR. For this reason, a thick slab of 8 mm was used. Future studies may explore optimizing the displacement encoding frequency for each subject to boost sensitivity and potentially reduce the slab thickness. The current study chose fixed encoding frequencies for each group (0.25 cyc/mm for IAA and DTA, and 0.17 cyc/mm for DAA) to allow even comparisons while preventing double-wrapping of the phase data. For the successful scans in this study, we note that the maximum non-smoothed displacement of any voxel for each group was 3.5 mm in the IAA, 4.4 mm in the DTA, and 4.5 mm in the DAA.

Furthermore, due to the loss of signal and increase in noise resulting from longitudinal through-plane motion, application of the current aortic DENSE CMR technique excluded the ascending aorta (where longitudinal strain can exceed circumferential strain [23]). In addition, since the calculation of 2D Green strain requires at least two pixels through the aortic wall and the current in-plane resolution is limited due to signal requirements, partial volume effects (particularly from surrounding structures with significantly different material properties like the vertebra) must also be carefully considered. The interested reader is directed to Wilson et al. [9], where these issues are discussed in more detail. In vitro validation of the quantification of strain by DENSE using a tubular polymer phantom in a CMR-compatible flow loop is also currently underway. We emphasize, therefore, that this current study evaluating novel aortic cine DENSE CMR in vivo is exploratory and that future larger studies are required both to confirm the independent influence of co-factors (e.g., sex and hypertension) and to strictly validate and quantify repeatability and reproducibility before formal clinical evaluation.

For this particular study, the analysis focused primarily on peak circumferential strain; however, the calculation of 2D Green strain from cine images also includes data from which radial strain, in-plane shear strain, non-peak strains, and strain rates could be calculated. Evaluation of these metrics will be reserved for future analyses, particularly since the radial and shear metrics are likely less reliable than the circumferential measurements due to the thinness of the aortic wall. Finally, future analyses using aortic DENSE should include quantification of current blood pressure, since the pulse pressure should be directly proportional to circumferential strain for any given patient. We note that in this control study, only a small number of subjects reported a history of hypertension. Furthermore, the normalization of the strain data also is expected to naturally reduce any variation due to differences in pulse pressure.

## Conclusions

This study provides four key insights regarding the patient-specific kinematics of the distal aortic arch, mid-descending thoracic aorta, and infrarenal abdominal aorta. First, each location demonstrated notable regional heterogeneities in circumferential strain around the aorta. Furthermore, when appropriately subdivided into subgroups (e.g., by age, displacement angle, etc.), the normalized strain distributions were remarkably consistent within each subgroup. We conclude, therefore, that normalization is a useful tool for interpatient comparisons of strain distributions and for the future study of the distinct effects that patient-specific factors have on local strain distribution (e.g., age, sex, hypertension). Second, the unique distributions of normalized circumferential strain when grouping by patient-specific factors emphasize the need for careful selection of control groups in future studies that aim to use patient-specific strain mapping to diagnose and/or assess the clinical risk of pathological aortic remodeling due to aneurysms, dissections, genetic anomalies, or other aortopathies.

Third, the spatial relationship of the regional distributions of circumferential strain to the mean displacement of the aorta and its surrounding structural supports (e.g., vertebra, pulmonary artery, etc.) highlight the importance of bulk motion of the aorta and peri-aortic constraints in determining patient-specific aortic kinematics. Thus, any computational models of aortic stress that assume homogeneous material properties, disregard subject-specific bulk motion of the aorta, and/or neglect spatially heterogeneous in vivo boundary conditions (i.e., peri-aortic constraints) must be interpreted with caution. Likewise, regionally heterogeneous kinematics within the aorta cannot be assumed to be due solely to differences in the material properties of the wall itself since external constraints may play a key role. This challenge must be considered in any future inverse methods to derive material properties from in vivo kinematics.

Finally, our results reveal unique kinematic properties in the DAA, including a significantly higher maximum NCS and heterogeneity index compared to the DTA, a change in the regional distribution of peak circumferential strain with increasing age, and the potential for highly elevated NCS in local diastole in patients with hypertension. The relevance of these unique findings to the propensity of aortic dissection in the DAA should be evaluated in future studies as aortic cine DENSE CMR is further developed and explored as a clinically useful tool for patient-specific diagnostics and prognostics.

## Data Availability

The datasets used and/or analyzed during the current study are available from the corresponding author on reasonable request.

## Abbreviations

AVI: Aorto-vertebral interface
BMI: Body mass index
BSA: Body surface area
bSSFP: Balanced steady state free procession
CAVI: Cardio-ankle vascular index
CMR: Cardiovascular magnetic resonance
CoV: Coefficient of variation
CT: Computed tomography
DAA: Distal aortic arch
DENSE: Displacement encoding with stimulated echoes
DTA: Descending thoracic aorta
ECG: Electrocardiogram
HASTE: Half-Fourier acquisition single-shot turbo spin-echo
IAA: Infrarenal abdominal aorta
IVC: Inferior vena cava
LA: Left atrium
LPA: Left pulmonary artery
LV: Left ventricle/left ventricular
NCS: Normalized circumferential strain
PC: Phase contrast
PWV: Pulse-wave velocity
SD: Standard deviation
TE: Echo time
TR: Repetition time
